# Supplementary material for: Acupuncture for Behavioral and Psychological Symptoms of Dementia: A Systematic Review and Meta-Analysis
Source: J Clin Med. 2021 Jul 13;10(14):3087. doi: 10.3390/jcm10143087 (PMC8303524; doi:10.3390/jcm10143087)
Supplement: Supplementary file 1 [file jcm-10-03087-s001.zip › jcm-1270699-supplementary/Supplement 2.pdf]

Supplement 2. Search terms used in each database

MEDLINE via PubMed

	Searches	Results
#1	Dementia[MeSH] OR dement*[Title/Abstract] OR Alzheimer*[Title/Abstract] OR “Lewy body”[Title/Abstract] OR Huntington*[Title/Abstract] OR Parkinson*[Title/Abstract] OR “Pick disease”[Title/Abstract] OR “cognitive impairment”[Title/Abstract]	399,150
#2	Acupuncture[MeSH] OR “Acupuncture Therapy”[MeSH] OR “Acupuncture Points”[MeSH] OR acupunct*[Title/Abstract] OR Electroacupuncture[MeSH] OR electroacupunct*[Title/Abstract] OR electro-acupunct*[Title/Abstract] OR acupoint[Title/Abstract]	32,330
#3	#1 AND #2	**800**

EMBASE via Elsevier

	Searches	Results
#1	'dementia'/exp OR 'dement*':ab,ti OR ‘Alzheimer disease’/exp OR ‘Alzheimer*’:ab,ti OR ‘Lewy body’/exp OR ‘Lewy body’:ab,ti OR ‘Huntington*’:ab,ti OR ‘Parkinson disease’/exp OR ‘Parkinson’:ab,ti OR ‘Pick disease’:ab,ti OR ‘cognitive impairment’:ab,ti	633,149
#2	‘acupuncture’/exp OR ‘acupunct*’:ab,ti OR ‘acupuncture point’/exp OR ‘electroacupuncture’/exp OR ‘electroacupunct*’:ab,ti OR ‘electro-acupunct*’:ab,ti OR ‘acupoint*’:ab,ti	51,549
#3	#1 AND #2	**1,237**

CENTRAL

	Searches	Results
#1	MeSH descriptor: [Dementia] explode all trees	5,964
#2	(dement* OR Alzheimer* OR “Lewy body” OR Huntington* OR Parkinson* OR “Pick disease” OR “cognitive impairment”):ti,ab,kw	34,558
#3	MeSH descriptor: [Acupuncture] explode all trees	151
#4	MeSH descriptor: [Acupuncture Therapy] explode all trees	4,749
#5	MeSH descriptor: [Acupuncture Points] explode all trees	2,002
#6	MeSH descriptor: [Electroacupuncture] explode all trees	803
#7	(acupunct* OR electroacupunct* OR electro-acupunct* OR acupoint):ti,ab,kw	16,846
#8	((#1 OR #2) AND (#3 OR #4 OR #5 OR #6 OR #7)) in Trials	**353**

AMED via EBSCO

	Searches	Results
#1	Dementia[SU] OR dement*[TX] OR Alzheimer*[TX] OR “Lewy body”[TX] OR Huntington*[TX] OR Parkinson*[TX] OR “Pick disease”[TX] OR “cognitive impairment”[TX]	6,788
#2	Acupuncture[SU] OR “Acupuncture Therapy”[SU] OR “Acupuncture Points”[SU] OR acupunct*[TX] OR Electroacupuncture[SU] OR electroacupunct*[TX] OR electro-acupunct*[TX] OR acupoint[TX]	11,781
#3	#1 AND #2	**129**

CINAHL via EBSCO

	Searches	Results
#1	Dementia[MH] OR dement*[TX] OR Alzheimer*[TX] OR “Lewy body”[TX] OR Huntington*[TX] OR Parkinson*[TX] OR “Pick disease”[TX] OR “cognitive impairment”[TX]	220,660
#2	Acupuncture[MH] OR “Acupuncture Therapy”[MH] OR “Acupuncture Points”[MH] OR acupunct*[TX] OR Electroacupuncture[MH] OR electroacupunct*[TX] OR electro-acupunct*[TX] OR acupoint[TX]	33,133
#3	#1 AND #2	**5,227**

PsycARTICLES via ProQuest

	Searches	Results
#1	SU(Dementia) OR ‘dement*’ OR ‘Alzheimer*’ OR ‘Lewy body’ OR ‘Huntington*’ OR ‘Parkinson*’ OR ‘Pick disease’ OR ‘cognitive impairment’	28,534
#2	SU(Acupuncture) OR SU(Acupuncture Therapy) OR SU(Acupuncture Points) OR ‘acupunct*’ OR SU(Electroacupuncture) OR ‘electroacupunct*’ OR ‘electro-acupunct*’ OR ‘acupoint’	350
#3	#1 AND #2	**117**

OASIS

	Searches	Results
#1	치매 AND 침	**4**

KISS

	Searches	Results
#1	치매 AND 침	**12**

RISS

	Searches	Results
#1	치매 AND 침	**2**

KMbase

	Searches	Results
#1	치매 AND 침	**5**

KCI

	Searches	Results
#1	치매 AND 침	**3**

CNKI

	Searches	Results
#1	(SU='痴呆'+'阿尔茨海默病'+'dementia'+'Alzheimer') AND (SU='精神行为'+'幻觉'+'妄想'+'攻击行为'+'焦虑'+'忧虑'+'烦乱'+'流浪'+'抑郁'+'失眠'+'睡眠'+'不寐'+'BPSD') AND (SU='针'+'鍼'+'acupuncture')	**77**

Wanfang data

	Searches	Results
#1	(主题:痴呆 or 主题:阿尔茨海默病 or 主题:dementia or 主题:Alzheimer) AND (主题:精神行为 or 主题:幻觉 or 主题:妄想 or 主题:攻击行为 or 主题:焦虑 or 主题:忧虑 or 主题:烦乱 or 主题:流浪 or 主题:抑郁 or 主题:失眠 or 主题:睡眠 or 主题:不寐 or 主题:BPSD) AND (主题:针 or 主题:鍼 or 主题:acupuncture)	**749**
