# Supplementary material for: Acupuncture for Behavioral and Psychological Symptoms of Dementia: A Systematic Review and Meta-Analysis
Source: J Clin Med. 2021 Jul 13;10(14):3087. doi: 10.3390/jcm10143087 (PMC8303524; doi:10.3390/jcm10143087)
Supplement: Supplementary file 1 [file jcm-10-03087-s001.zip › jcm-1270699-supplementary/Supplement 3_revised.pdf]

Supplement 3. Excluded studies after full-text review

1) not clinical study: 3

1. Cobb, K. Acupuncture and Alzheimer's Disease. *Acupuncture Today* **2020**, *21*, 44-47.
2. Zhu, M.; Zhao, L. Professor Zhang Hong's experience in the treatment of vascular dementia with electroacupuncture on the scalp points. *Lishizhen Medicine and Materia Medica Research* **2009**, *20,* 2581-2582, doi:10.3969/j.issn.1008-0805.2009.10.099.
3. Han, J.; Yu, J.; Kan, B.; Zhang, X.; Cao, X.; Luo, B.; Jia, Y. Research on G protein signal transduction pathway of acupuncture treatment of Alzheimer's disease. *Tianjin Journal of Traditional Chinese Medicine* **2012**, *29*, 68.

2) only abstract available without raw data: 5

1. Colling, C.; Holthoff-Detto, V.; Falkai, P.; Musil, R. NADA-Auriculoacupuncture as a Symptomatic Treatment for Agitation in People with Dementia: A Randomized, Controlled, Rater-Blind Pilot Study. *Journal of Acupuncture & Meridian Studies* **2018**, *11*, 243-244, doi:10.1016/j.jams.2018.08.164.
2. Willcox, T.; Yang, J.; Galvin, J.; Rosenfeld, A.; Tolea, M.; Fan, Y.; Cao, J. Observation on the efficacy of acupuncture in the treatment of mild cognitive impairment (MCI) caused by Alzheimer's disease (AD) in the elderly. In Proceedings of New Era, New Thinking, New Leap, New Development-2019 China Acupuncture and Moxibustion Society Annual Conference and 40th Anniversary Review, Wuhan, Hubei, China; p. 7.
3. Zhao, C.T.; Luo, J.G.; Yin, H.Y.; Luo, L.; Zeng, F.; Yu, S.G. Acupuncture for behavior and psychological symptoms of dementia: phase I clinical trial. *Journal of alternative and complementary medicine.* **2013**, *19*, A18, doi:10.1089/acm.2013.1502a.
4. Yu, S.; Ying, J.; Zhang, H.; He, Y.; Tang, Y. Acupuncture Treatment of 30 Cases of Senile Dementia with Mental and Behavioral Symptoms. In Proceedings of Chinese Academy of Acupuncture and Moxibustion The 7th National Symposium on Acupuncture and Tuina for Young People, Lhasa, Tibet, China; p. 4.
5. Kami, H.; Hiroshi, K.; Tsedeng, T.; Heto, M. A case of triple-focus acupuncture for a patient with dementia. In Proceedings of the 10th Beijing International Rehabilitation Forum, Beijing; pp. 431-432.

3) not report diagnostic criteria of dementia: 3

1. Kwok, T.; Ip, I.; Wing; Leung, P.-C.; Ho, F.; Wong; Ho; Wong. The effectiveness of acupuncture on the sleep quality of elderly with dementia: a within-subjects trial. *Clinical Interventions in Aging* **2013**, *2013*.
2. Wen, M.; Cheng, H.; Shang, L.; Liu, Y.; Yu, J. One case of Alzheimer's disease mental and behavioral disorder treated by Prof. Han Jingxian's conditioning of triple focus. *Journal of Clinical Acupuncture and Moxibustion* **2012**, *28*, 22-23, doi:10.3969/j.issn.1005-0779.2012.08.014.
3. Zhao, C.; Luo, J.; Ren, Y.; Tang, Y. The experience of using "ghost point" acupuncture to relieve the mental behavior symptoms of Alzheimer's disease. *Nei Mongol Journal of Traditional Chinese Medicine* **2011**, *30*, 65-66.

4) not about acupuncture: 1

1. Yu, Y.; Zhang, J.; Du, L.; Zhou, J.; Jiang, X.; Zheng, Y. Clinical observation of acupoint pointer intervention in patients with sleep disorders in Alzheimer's disease. *Prevention and Treatment of Cardio-Cerebral-Vascular Disease* **2018**, *18*, 74-76, doi:10.3969/j.issn.1009_816x.2018.01.025.

5) comparison of different acupuncture: 2

1. Li, X. To Observe The Clinical Effect of "Needling Anshen Point Group" in The Treatment of Senile Dementia of Sleep Disorders. Master's degree, Shandong University of Traditional Chinese Medicine, 2017.
2. Mo, F.; Li, J.; Deng, T. The comparison of clinical results in treating vascular dementia with laser radiation and acupuncture. *J trad chin med* **2001**, *36*, 353.

6) not report outcome of interest: 18

1. Chen, Z.F.; Jiang, G.H.; Lai, X.S. Clinical observation on the electronic acupuncture treating vascular

dementia. *Journal of clinical acupuncture and moxibustion* **2000**, *16*, 18-20.
2. Dong, H.-T.; Jin, Y.-G.; Au, Y. 11 Cases of Alzheimer 's Disease Treated by Acupuncture. *Acta universitatis traditionis medicalis sinensis pharmacologiaeque shanghai* **2002**, *16*, 26.
3. Huang, W.C.; Xu, J.X.; Wang, X. Study on the acupuncture in treating multiple infarctional dementia. *Zhongguo zhen jiu [chinese acupuncture & moxibustion]* **1992**, *12*, 1-3.
4. Jiang, G.H.; Chen, Z.H.; Lai, X.S. Clinical study on treatment of vascular dementia with electroacupuncture of 'sishenchong'. *[world journal of acupuncture-moxibustion]: world j acup-moxi* **2003**, *13*, 10-13.
5. Lain, X.S.; Zhang, J.W.; Muo, F.Z.; Jiang, G.H.; Li, J.Q.; Zheng, Z.C. Clinical observation on the effects of acupuncture in the treatment of vascular dementia. *Chinese acupuncture and moxibustion* **1997**, *12*, 713-716.
6. Liu, H.A.; Hou, D.F.; Diao, Z.Y.; Wang, Y. Observation on the clinical curative effects of turbit clearing and intelligence-improving acupuncture therapy on vascular dementia and the study onits mechanisms. *Zhongguo zhen jiu [chinese acupuncture & moxibustion]* **1997**.
7. Liu, J.; Peng, X.H.; Lin, D.D.; Li, C.D.; Jiang, Z.Y.; Zeng, L. Clinical study of electrical scalp acupuncture in the treatment of vascular dementia. *Chinese acupuncture and moxibustion* **1998**, *4*, 197-200.
8. Lun, X. Clinical observation of dementia treated by surrounding-acupuncture on CT location. *Tcm shanghai journal of acupuncture and moxibustion* **2000**, *3*, 40.
9. Lun, X.; Feng, B.-F.; Rong, L. Clinical observation of vascular dementia treated by surrounding-acupuncture of the ct-located area. *J acu tuina sci* **2003**, *1*, 20.
10. Mo, F.-Z. The influence of electroacupuncture on intelligence structure in patients with vascular dementia. *Shanghai journal of acupuncture and moxibustion* **2001**, *20*, 6.
11. Mu, Y. The Treatment of Alzheimer's Disease by the Combination of Acupuncture and Medicine. *Shanghai journal of acupuncture and moxibustion* **2001**, *20*, 3.
12. Shi, G.X.; Li, Q.Q.; Yang, B.F.; Liu, Y.; Guan, L.P.; Wu, M.M.; Wang, L.P.; Liu, C.Z. Acupuncture for Vascular Dementia: A Pragmatic Randomized Clinical Trial. *TheScientificWorldJournal* **2015**, *2015*, 161439, doi:10.1155/2015/161439.
13. Shi, G.X.; Liu, C.Z.; Li, Q.Q.; Zhu, H.; Wang, L.P. Influence of acupuncture on cognitive function and markers of oxidative DNA damage in patients with vascular dementia. *Journal of traditional Chinese medicine = Chung i tsa chih ying wen pan* **2012**, *32*, 199-202, doi:10.1016/s0254-6272(13)60011-4.
14. Wang, Y.; Qin, W.G.; Yu, C.D. Clinical observation on effect of cranial suture acupuncture combined with donepezil hydrochloride tablets for Alzheimer's disease. *World Journal of Acupuncture - Moxibustion* **2014**, *24*, 19-24, doi:10.1016/S1003-5257(14)60020-9.
15. Zhao, L.; Zhang, H.; Zheng, Z.; Huang, J. Electroacupuncture on the head points for improving gnosia in patients with vascular dementia. *Journal of traditional Chinese medicine = Chung i tsa chih ying wen pan* **2009**, *29*, 29-34, doi:10.1016/s0254-6272(09)60027-3.
16. Lin, T.; Guo, X.; Huang, H.; Wang, H.; Liang, S. Clinical Study on Electroacupuncture Treatment of Cognitive Impairment in Cerebral Infarction. *World Chinese Medicine* **2010**, *5*, 125-126, doi:10.3969/j.issn.1673-7202.2010.02.027.
17. Yang, H.; Li, X.; Guo, Z.; Chen, X. The effect of scalp needle extraction on the mental state of patients with vascular dementia with mental and behavioral abnormalities. *Zhejiang Journal of Traditional Chinese Medicine* **2017**, *52*, 689, doi:10.3969/j.issn.0411-8421.2017.09.042.
18. Yu, X. Acupuncture treatment of vascular dementia with the method of strengthening the supervision and strengthening the brain and regulating the mind. *Journal of Clinical Acupuncture and Moxibustion* **2011**, *27*, 22-23, doi:10.3969/j.issn.1005-0779.2011.11.010.

7) use duplicate data: 4

1. Lombardo, E.N.; Vehvilainen, L.; Ooi, W.L.; Volicer, L.; McManus Cet, a. Acupuncture to treat anxiety and depression in Alzheimer's Disease and vascular dementia: a pilot feasibility and effectiveness trial. *Proceedings of the world alzheimer congress; 2000 jul 9-13, washington , DC* **2000**.
2. Lombardo, N.B.E.; Dresser, M.V.B.; Malivert, M.; McManus, C.A.; Vehvilainen, L.; Ooi, W.L.; Xu, G.;

Rosowsky, E.; Drebing, C.; Sheridan, P.L., et al. Acupuncture as treatment for anxiety and depression in persons with dementia: results of a feasibility and effectiveness study...Reprinted with permission from Alzheimer's Care Quarterly. *Journal of the Acupuncture Association of Chartered Physiotherapists* **2009**, 45-59.

3. Zhao, C. Acupuncture at ghost points for the treatment of mental and behavioral symptoms of Alzheimer's disease. Phase I clinical trial. Master's degree, Chengdu University of Traditional Chinese Medicine, 2011.
4. Lu, Y.Y.; Huang, D.T.; Liao, L.L.; Huang, H. Clinical Study on Cluster Needling of Scalp Point Therapy for Depression Caused by Alzheimer's Disease. *Journal of Clinical Acupuncture and Moxibustion* **2015**, 10.3969/j.issn.1005-0779.2015.04.012, 29-31, doi:10.3969/j.issn.1005-0779.2015.04.012.

8) not available full-text: 1

1. Chen, B. Clinical observation on acupuncture treatment of cerebrovascular dementia - a report of 32 cases. *Journal of Traditional Chinese Medicine* **2001**, *21*, 174-176.
